# Mating Market and Dynamics of Union Formation

**DOI:** 10.1007/s10680-021-09592-2

**Published:** 2021-09-21

**Authors:** Giulia Corti, Stefani Scherer

**Affiliations:** 1 School of Social Sciences, University of Trento, Trento, Italy; 2 Department of Statistical Sciences “Paolo Fortunati”, University of Bologna, Bologna, Italy

**Keywords:** Assortative mating, Mating market, Union formation, Reversed gender gap in education, Hypogamy

## Abstract

The paper investigates the relationship between structural partner market constraints and the timing and educational sorting of unions in Germany (1985–2018). We integrate the literature on the effect of the reversed gender gap in education on educational assortative mating, with a focus on mating dynamics and the measurement of the partner market over the life course. We concentrate on two particular educational groups, low-educated men and highly educated women, those with worsening mating prospects and more subject to experience hypogamous unions. Our results show that the local education-specific mating squeeze influences union formation, its timing, and educational sorting. Indeed, for the two groups, the increasing supply of highly educated women in the partner market increases the likelihood of remaining single or establishing an hypogamous union, where she is higher educated than he. In line with search theory, we find the effects of the mating squeeze to become particularly visible after people turn 30 years of age. This is true for the risk of remaining single and forming an hypogamous union. We underline the necessity to study assortative mating and union formation from a dynamic perspective, taking into account changing structural conditions during the partner search process.

## Introduction

Assortative mating is a key characteristic of couple formation and concerns various traits like ethnicity, religion, age, or physical appearance (Kalmijn, 1998; Schwartz, 2013). Educational assortative mating has received extensive attention because it is an important element for the unequal distribution of resources between households (Breen & Salazar, 2011; Mare & Schwartz, 2006; Schwartz, 2013), as education influences an individual’s earnings, occupation, and life chances, as well as being a good indicator of values, aspirations, and lifestyle (Blossfeld, 2009; Blossfeld & Timm, 2003). The changes of patterns of educational assortative mating come with potential consequences not least in the reproduction of inequalities through couple formation (Mare & Schwartz, 2006; Schwartz, 2013).

Mating not only depends on preferences but is also linked to the structural composition of the population and the availability of potential partners. Since the 1970s, women’s involvement in education has steadily expanded, until women overtook men in educational attainment at the beginning of the twenty-first century in Western countries (Esteve et al., 2012). This process is supposed to influence the structure of the partner market, changing the number of potential partners across educational groups. In particular, the growing supply of higher educated women relative to men is known to have raised a «new education-specific mating squeeze» (De Hauw et al., 2014), which in turn is expected to influence mating behaviors.

Research documented a general postponement in union formation, increasing rates of singlehood (Bellani et al., 2017), and changes in traditional patterns of educational assortative mating with a rise in couples in which the woman is more educated than the man (hypogamous) relative to those in which the man holds a higher educational degree (hypergamous) (Esteve et al., 2016). An association between women’s growing involvement in higher education and the rise of hypogamous couples has been documented both at the aggregate and individual level (Esteve et al., 2016; Van Bavel et al., 2018), but less research has dealt with the dynamic nature of this relationship and its timing across the life course. Moreover, understanding the dynamics of couple formation might provide pieces of understanding about their consequences on other family-related aspects such as fertility decisions (Nitsche et al., 2018; Trimarchi & Van Bavel, 2019), couple instability, and the division of paid and unpaid labor within the couple (Grow et al., 2017; Theunis et al., 2018).

The paper analyses the role of (changing) partner market conditions for the timing of union formation (cohabitation and marriage) and educational assortative mating in Germany, with a particular interest in the occurrence and timing of hypogamy, adopting a dynamic life-course perspective. We test if and to what extent conditions of the partner market influence couple outcomes differently according to age. We expect structural constraints to become more important as individuals age.

We provide two contributions. First, we study the timing of union formation and assortative mating jointly, and we do so by distinguishing different groups. A partner market squeeze likely affects educational groups and sexes unequally. Following the literature (Van Bavel, 2012), we focus on two groups that might face particularly adverse mating market conditions due to structural reasons: low-educated men and highly educated women, providing insights of diversified behavior. Second, we adopt a dynamic life-course perspective investigating how the effects of the squeeze change with age. Following search theory (Oppenheimer, 1988), the (changing) structural conditions of the mating market should become increasingly relevant for educational sorting if the partner search process gets longer. Therefore, we measure the partner market situation from a dynamic perspective, taking into account specific age-related dynamics and test new measures that vary as individuals grow older (Skopek et al., 2011).

Using GSOEP data, we follow individuals born between 1961 and 1990 in their partner search process (might that end in a cohabitation or marriage). The focus lies on the (changing) effects of the regional partner market situation over time and on the changing importance of these structural constraints as people age. Results from event history models demonstrate that the structural conditions of the partner market in terms of a growing supply of highly educated women come with implications for the dynamics of union formation, increasing the chances of both singlehood and hypogamy.

## Theoretical Background

### The Mating Market

The vast literature on assortative mating has identified three main drivers of partner choice (Kalmijn, 1998; Schwartz, 2013): third parties, such as parents, peers, and the institutions in which an individual is embedded (Kalmijn, 1998), individual preferences and structural constraints. Individuals do have preferences for a partner in terms of a set of given characteristics that guide the search. As for educational matching, individuals have been found to prefer partners with similar educational attainment, also because education is a good predictor of values and lifestyles (Blossfeld, 2009; Kalmijn, 2013). Moreover, the partner search takes place under structural constraints in terms of the composition of the population. This is commonly known as the mating market, which can be identified as the physical and symbolic place in which individuals face a situation of availability and competition for potential partners with mating-relevant characteristics such as gender, age, education, ethnicity, and values (Kalmijn, 1998). Indeed, demographic, social, and economic factors can influence population characteristics and create an imbalance in the mating market. The general idea underlying the notion of the mating market is that the presence (or absence) of eligible partners influences partnership formation (Choi & Tienda, 2017; De Hauw et al., 2014; Lichter et al., 2019; Qian & Lichter, 2018; Schoen, 1983).

The literature on the mating market traditionally focused on sex ratios as determinants of union formation. Unbalanced sex ratios have been a matter of concern in many historical circumstances (Abramitzky et al., 2011; Angrist, 2002; Guilmoto, 2012; Jiang et al., 2014), and it has been found that union formation opportunities are depressed when faced with a shortage of (suitable) potential partners (Akers, 1967; Angrist, 2002; Fossett & Kiecolt, 1991). The structuralist perspective has extended this reasoning beyond the raw balance between men and women and proposes a more explicit sociological focus on social heterogeneities and the size of variously defined social groups as a determinant of union formation and assortative mating (Blau et al., 1982).

Further, the mating market influences also the dynamics of the partner search process, as proposed by Oppenheimer (1988). Following the ideas of job search theory, individuals seek a partner from a distribution of potential mates about which they have imperfect information. This search entails some costs related to time investment and emotional risks. The benefits refer to achieving a well-matched partnership. In analogy to the reservation wage of job seekers below which an offer is not accepted, in the partner search process there are also a set of required minimum characteristics below which the search will continue. However, the costs related to partner search are not static over the life course but are continuously redefined as individuals age and their value in the mating market changes, which has implications for the kind of match accepted. Empirical applications of the search theory in the US have shown that unfavorable marriage markets increase the chances of hypogamy, especially at older ages (Lewis & Oppenheimer, 2000).

### Mating Squeeze and Micro-level Dynamics

Educational homogamy has traditionally been and still is the rule in educational assortative mating (Blossfeld, 2009). Recently, a new tendency for heterogamous couples emerged, with hypogamous couples becoming more common than hypergamous ones among younger cohorts (Esteve et al., 2012, 2016), but at the same time, Chudnovskaya & Kashyap (2020) show hypogamous couples to be negatively selected on some characteristics.

Among the reasons for these changing patterns, structural factors like shifts in the composition of the mating market, more specifically in the distribution of educational attainment of men and women, likely play a key role.^1^ Since the 1970s, Western countries have witnessed a steady increase in women’s participation in higher education (OECD, 2019) to a point in which women outnumber men among the higher-educated, thus reversing the traditional male advantage in education (Buchman et al., 2008). Social scientists refer to this phenomenon as the reversed gender gap in education (Van Bavel, 2012; Van Bavel et al., 2018), and it has been directly linked to the occurrence of a new education-specific mating squeeze (De Hauw et al., 2014; Van Bavel, 2012).

As for the consequences of the occurrence of a mating squeeze on union formation patterns, some studies document an association between the rise of hypogamy and the increasing advantage of women in higher education in European and extra European countries (Esteve et al., 2012, 2016). At the individual level, De Hauw et al. (2017) showed that in Europe the reversed gender gap in education is associated with a higher likelihood of hypogamy across cohorts for tertiary educated women, although no effect is found for singlehood. Conversely, low-educated individuals—both men and women—are found to be more at risk for singlehood with the increasing advantage of women in tertiary education. However, this study relies on cross-sectional data and is thus not able to account for the dynamic nature of the search process and the complex interaction between age and mating market constraints. Further, it heavily relies on between-country comparisons which likely makes results subject to bias due to unobserved factors. Other studies (Eckhard & Stauder, 2018) adopt a life-course perspective in the measurement of the partner market and its impact on the union formation process in Germany, where education-specific squeeze measures appear to be poor predictors for the probability of union formation. These contributions focus mainly on methodological aspects about the measurement of the partner market.

The literature pointed out two educational groups that might experience a progressive shortage of potential equally educated partners in the mating market (De Hauw et al., 2017; Van Bavel, 2012). Women with tertiary education are expected to deal with a shortage of equally educated potential partners as a direct effect of the growing supply of highly educated women. At the same time, women’s educational expansion diminishes the group of low-educated women, thus creating a shortage of potential equally educated partners for low-educated men and making them relatively more disadvantaged in the partner market. Whereas research has traditionally focused on women with tertiary education (Kalmijn, 2013), less attention has been devoted to men. However, nowadays, the highest rates of singlehood are found among low-educated men (Bellani et al., 2017), and it is possible to expect that recent changes in union formation patterns would involve, and possibly change, men’s traditional position in the couple. Thus, the investigation of men is particularly interesting (Van Bavel, 2017).

## Research Question and Expectations

This paper investigates the role of (changing) conditions in the partner market for union formation process. More precisely, we study to what extent the rise of an education-specific mating squeeze stemming from the growing involvement of women in tertiary education shapes the timing and patterns of educational assortative mating. We focus on low-educated men and highly educated women in Germany but provide analyses for the other groups in “Appendix B”.

We investigate the role of structural changes. Following search theory (Oppenheimer, 1988), we expect partner market characteristics to be relevant for union formation, and their role to vary over the life course. Benefits and costs of partner search evolve with age, and coping with unfavorable mating conditions can be considered as part of the costs. At younger ages, facing a shortage of desired potential partners could be affordable, because postponement is a viable solution. However, the longer the search and the closer it gets to the normative ages for finding a partner (Wrosch & Heckhausen, 1999)—usually around the thirties—the higher the costs of prolonging the search. Therefore, the influence of mating market structure should become more relevant for couple formation as persons age. This implies that tighter mating market conditions will negatively influence entering a union especially as persons reach an age around 30 (Hypothesis 1).

Further, structural constraints are also expected to be relevant for educational sorting. Extending search theory by mechanisms from the psychological literature, we can expect individuals to adjust their expectations as family formation becomes more salient (Spielmann et al., 2013), especially if the availability of partners is structurally limited. In terms of educational sorting, this implies that a generally less desired educational outcome such as hypogamy might become more acceptable with the aim to avoid singlehood.^2^ Thus, we expect a tighter mating market to come with an increase in the likelihood of hypogamy especially as persons age (Hypothesis 2).

## The German Context

**Fig. 1:**
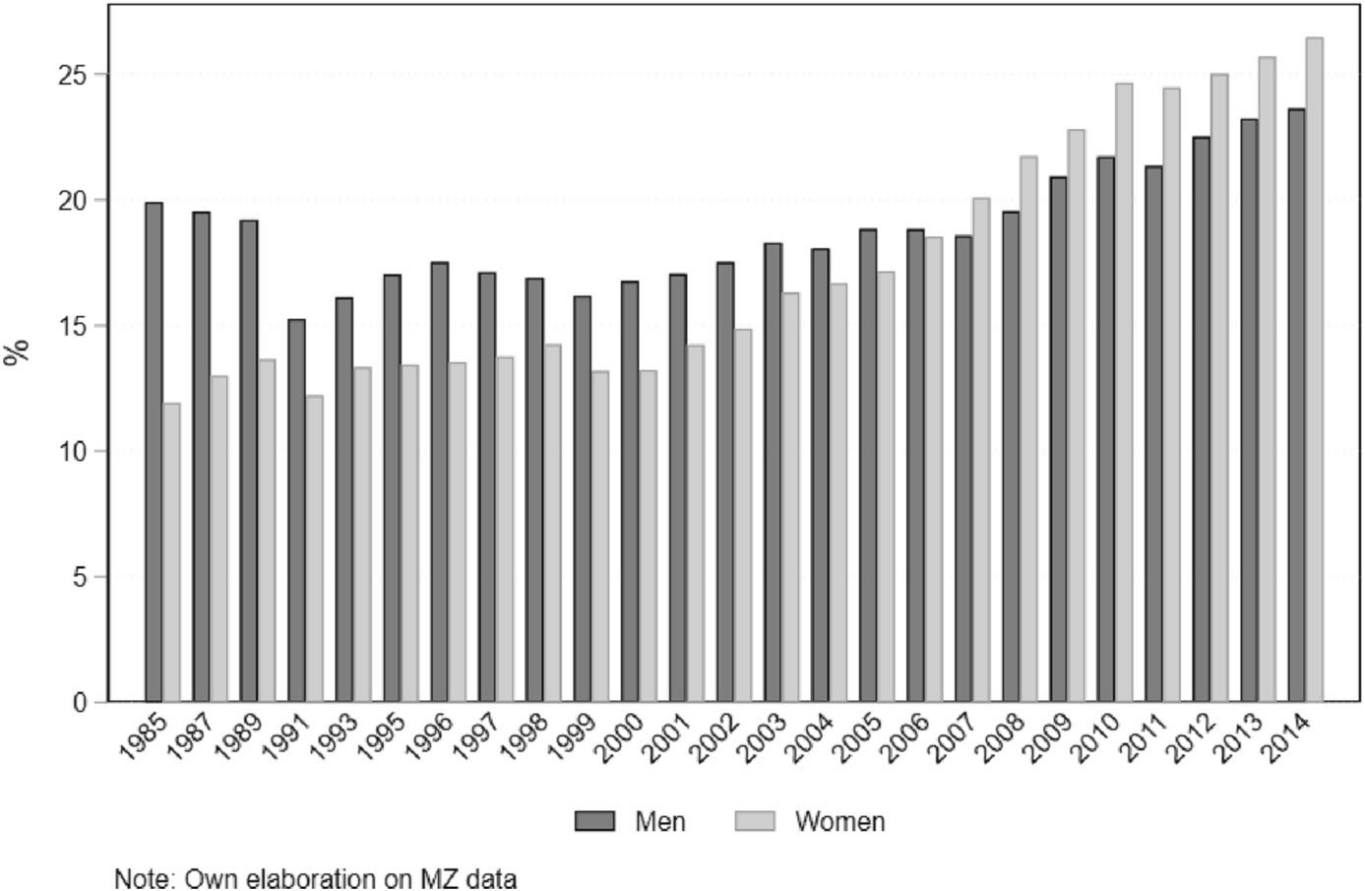
Share of tertiary educated individuals at the age 30–34, Germany

Compared to other Western countries, in Germany the advancement of women in higher education has followed a slower pace. Whereas already at the beginning of the 2000s most Western societies had experienced a reversing in the traditional male advantage in higher education, Germany, together with Austria and Switzerland, was behind (De Hauw et al., 2017). Women started outnumbering men in higher education only in 2007 (Fig. 1). Among the reasons is the fact that only in the late 2000s access to universities has been given also to students from vocational training programs (Triventi, 2014).

Also, social change regarding demographic transitions started relatively late in Germany compared to other Western countries, and still today some trends are less evident, as it has been extensively documented in the literature (Billari & Liefbroer, 2010; Buchmann & Kriesi, 2011; Köppen, 2011). In line with a general trend, the age at first union formation has increased across cohorts, especially for marriage: the age at first union passed from 22 years for the 1945–1954 cohorts to 24 for the 1970s cohort, and the age at marriage increased from 22 to almost 28 (Köppen, 2011). Furthermore, the proportion of marriages that began as non-marital cohabitation increased over time. Whereas in the 1960s, 10% of couples started as a cohabitation, and this share increased up to 74% in the 1990s (Köppen, 2011). These changes have followed a strong educational gradient, with higher individuals being the forerunners in adopting new behaviors (Köppen, 2011). As for trends in educational assortative mating, it has been documented a steady increase in hypogamous couples across cohorts, even though with a slower pace compared to other European countries (De Hauw et al., 2017).

## Data, Measures, and Analytical Strategy

### Data

We reconstruct the detailed process of couple formation and look at the dynamics of union formation and educational sorting. Analyses are based on G-SOEP data for the years 1985–2018, a period long enough to observe birth cohorts from 1961 to 1990. Our analytical sample of 4,396 individuals (37,806 observations) involved in the partner search process is composed of individuals who entered the observational window as single, not previously married, and between 20 and 30 years of age. On average, individuals enter the sample at age 24 and are observed for 7.7 years (see “Appendix A”, Table 1 for sample characteristics). In the robustness checks, we confine the sample to the native population, but the results do not differ substantively. In line with this, also controlling for immigration status in the models does not alter the results.

To construct the mating market measures, we used German Micro Census (MZ) data,^3^ which provide information on the number of women and men across all levels of education by year, regions (Länder), and age groups.^4^

### Measures

#### Union Formation and Educational Assortative Mating

The union formation process is observed considering jointly the information on singlehood and educational sorting of the partnership, independently of whether it is cohabitation or marriage. We distinguish the following states: singlehood, homogamy (partners have the same education), hypogamy (woman more educated than the man), and hypergamy (man more educated than the woman).^5^ The educational attainment of individuals and their partners is operationalized as low (lower than secondary), medium (upper secondary), and high level (tertiary education).

#### Mating Market

Our main explanatory variable accounts for the structure of the mating market. We combine the mentioned streams of the literature in the construction of our mating market measures. We keep the notion of sex ratios, which give a measure of the number of women relative to men, and we follow the suggestions coming from the structuralist theory and more recent literature (De Hauw et al., 2014), focusing on the size of a particular social group—highly educated—among the categories of interest such as age, region, and marital status.

The pool of potential partners includes individuals 2 years younger or older than the individual. The preference for a potential partner with similar age should be likely due to the preference for individuals with similar life experiences, tastes, values, and aspirations (Skopek et al., 2011). Indeed, when asked about age preferences for a partner, most individuals indicate similar ages to their own (Ni Bhrolcháin, 2001; Skopek et al., 2011). Moreover, recent studies on online dating suggest that in a setting in which age can be applied as an explicit sorting criterion, age assortativity increases (Potarca, 2020; Thomas, 2020).

As for the geographical scope of the partner market, preferences for geographical proximity and contact opportunities suggest restricting markets geographically. However, the recent technological and transport development extended the scope beyond the place of residence. We delimit the geographical horizon of our partner market measures at the regional level, the German Länder.^6^

The market measure includes both single and married individuals, for two main reasons. First, using measures based just on singles might introduce some endogeneity issues (De Hauw et al., 2014). Indeed, such measures are by construction affected by rates of union formation and dissolution, and using them as an independent variable to study union formation introduces a reversal causal effect. Secondly, empirical results have shown that partner market measures with singles are highly correlated with those with also married people, thus making not necessary to include just singles (Fossett & Kiecolt, 1993; Cready et al., 1997; Albrecht et al., 1997; Angrist, 2002; Albrecht & Albrecht, 2001; Grossbard & Ameudo-Dorantes, 2007).

The composition of the mating market is not stable but varies over cohorts and with individuals’ age. We thus propose partner market measures that are updated annually.

Based on these considerations, we construct a further development of the measure of the gender balance in higher education proposed by De Hauw et al. (2017), driven by the fact that the most relevant changes in the educational distribution have occurred in the access to tessrtiary education. The operationalization is the following:

1 $${R}_{i}= \frac{{\sum }_{k-2}^{k+2}{W}_{h=3tr}}{{\sum }_{k-2}^{k+2}{M}_{h=3tr}}$$

The gender balance in higher education is the ratio between the total number of women *W* with a tertiary degree *h* = *3* over that of men *M* in an age range *k* ± *2* than the individual *i*, in each year *t* and each region *r*. For instance, for an individual aged 26 in Bavaria in a year *t*, the corresponding measure of the mating market is the ratio between the number of women and men with higher education who are between 24 and 28 years of age residing in the same region in that year. As the individual turns 27, his/her market situation is updated with the ratio of tertiary educated women and men between 25 and 29, and so on as the individual gets older. This measure of the squeeze, identical for men and women, varies across local mating markets over historical time and with individuals’ age. With values less than one, more men than women are higher educated, whereas values greater than one indicate that women have an advantage instead.

Furthermore, women’s and men’s age preferences might evolve differently during the life course (Skopek et al., 2011). Whereas men increase their preferences for younger partners with age, women are shown to adapt their preferences as they advance in their life course. This could come with consequences in sorting. If men have an enlarged pool of potential partners, they should have higher chances to find an equally educated woman. On the other hand, it has been shown how women might become more disadvantaged in the partner market (Skopek et al., 2011). Whereas they look increasingly for similar-aged men as they age, they are less favored by men of their same age group. This could increase the likelihood of singlehood, but also that of hypogamy; a less desired trait such as the woman’s age could be exchanged with the potential partner's educational level. We account for these differences by building a second measure of gender balance in higher education with mobile age barriers where we allow the age boundaries to enlarge during the life course for men. In this measure, which we use just for men, we include in the pool of potential partners and competitors individuals of younger ages starting from 25 years of age onwards. For instance for a man who is 33 the pool of potential partners and competitors will span from 25 to 35 years of age.

### Analytical Strategy

We first describe trends in mating market conditions across regions over time and union formation and educational assortative mating patterns. The main analyses test whether and to what extent our measures of mating squeeze are associated with the transition to partnership and educational assortative mating. We apply discrete-time event history models (Allison, 1984), with multinomial specification specifying the following model:

2 $${h}_{itr}= \alpha {D}_{it}+ \beta edu+ \beta squeeze+ \beta cohort+ \beta squeeze*{D}_{it}+ {\upgamma }_{r}$$

where *D*_*it*_ refers to a set of dummy variables for age groups (with 20–22 as the reference category) that determine the shapes of the hazard, *βedu* refers to education and *βcohort* represents the coefficient for the birth cohorts. To control for unobserved region-specific factors, we include *γ*_*r*_ region fixed effects. Since we aim to test the age-related dynamics of mating market conditions, the model includes an interaction term between age and market squeeze. We run separate models for men and women with standard errors clustered at the individual level.

## Results

### Trends in Mating Market Composition

**Fig. 2:**
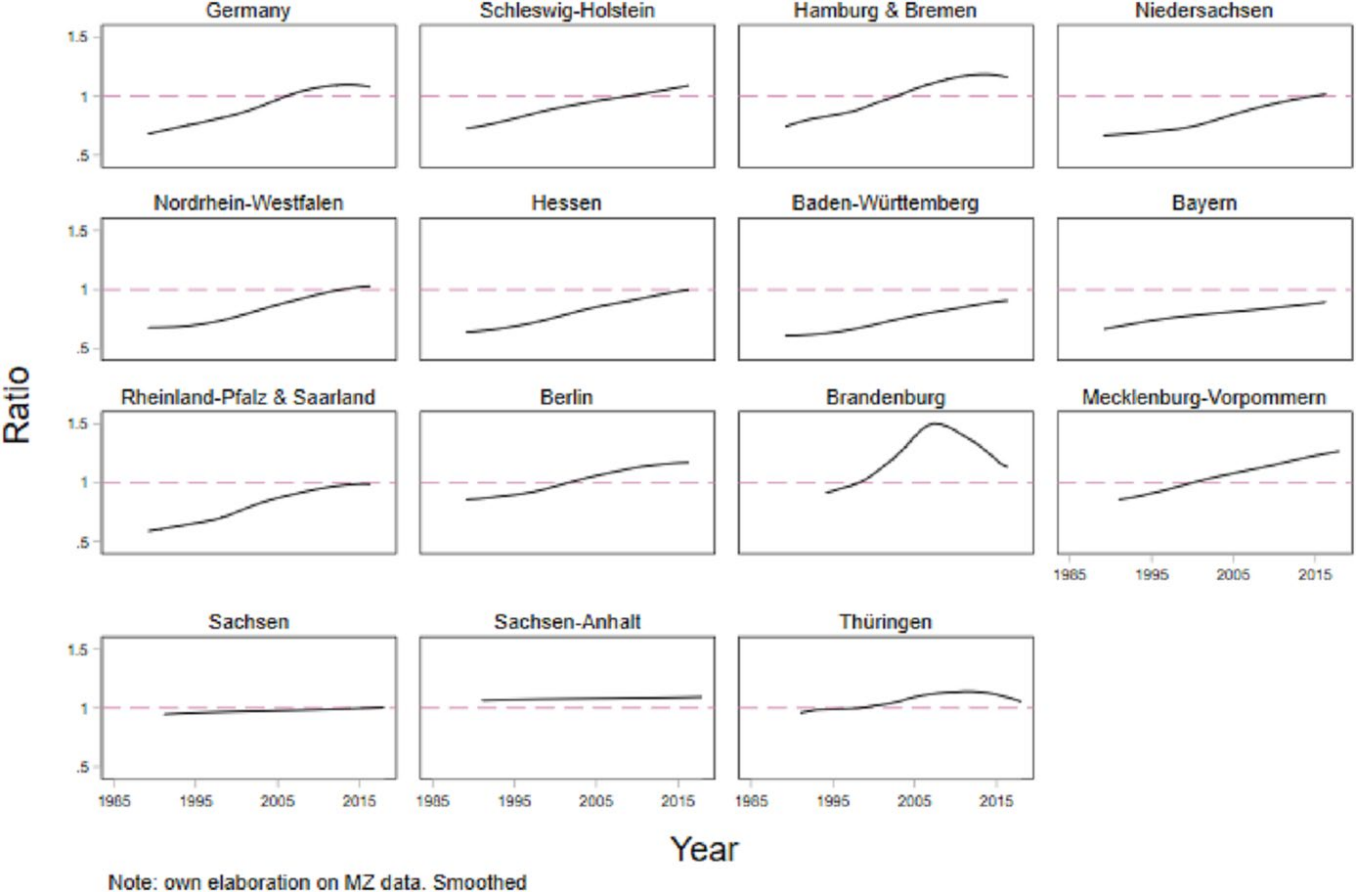
Educational mating squeeze at the regional level, 1985–2018. Ratio of high educated women over high educated men at age 30–34

Figure 2 documents trends over time in the educational composition of the regional mating market and displays the trend of the ratio between women and men between 30 and 34 years with a tertiary degree in German regions. There is a clear trend toward an educational mating squeeze, but it is not homogeneous. Relevant differences in the pace and the timing of the reversed gender gap between regions are visible. Whereas some regions—such as Hessen, Bavaria, Lower Saxony, and Rhineland-Palatinate with Saarland—have recently experienced this turn-out, others, mainly the city-states (i.e., Berlin, Hamburg & Bremen) have a stronger positive trend from earlier years.

### Trends in Union Formation and Educational Assortative Mating

**Fig. 3:**
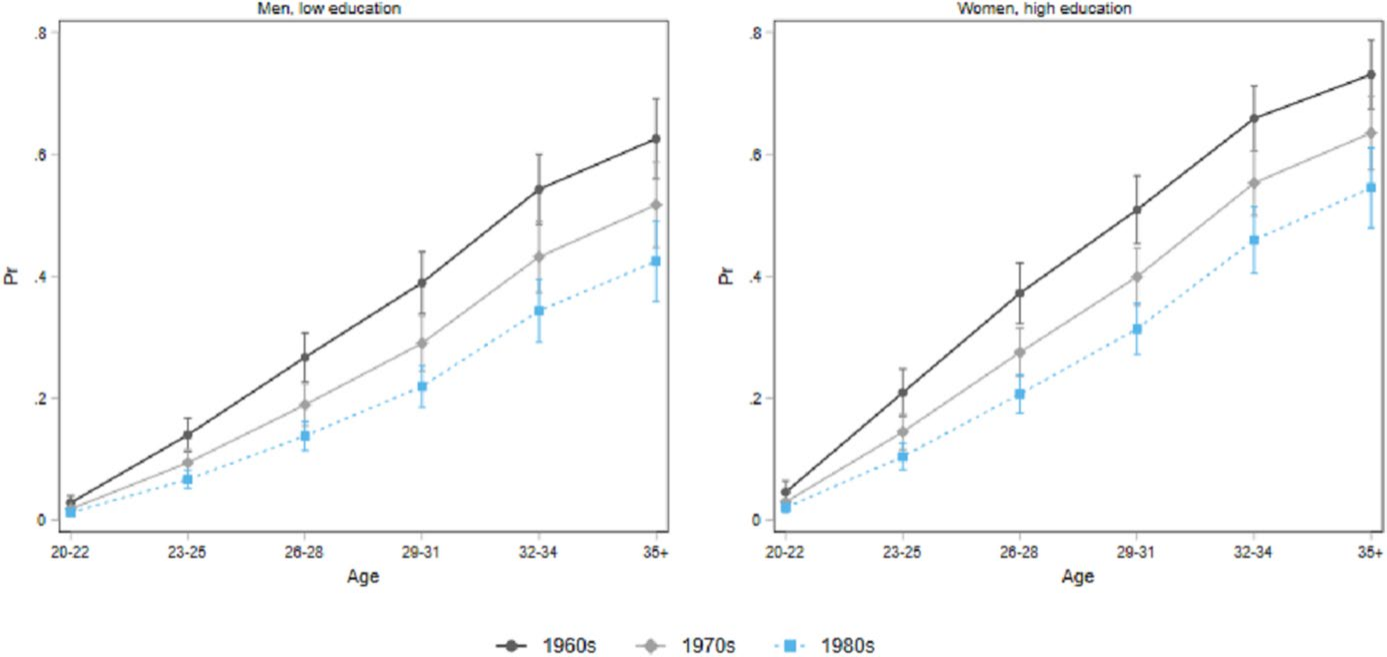
Hazard ratios of being in a relationship, men with low education and women with high education by birth cohorts

Figure 3 describes the development of the hazard ratio of union formation (defined as entering a cohabitation or marrying) with age for low-educated men and highly educated women from the cohorts born in the 1960s, 1970s, and 1980s in our sample. Low-educated men have a higher risk of remaining single compared to higher educated women, showing a pattern of stronger disadvantage, that becomes more pronounced among younger cohorts. Thus, in the first step of selection into union, low-educated men appear those with greater disadvantage.

**Fig. 4:**
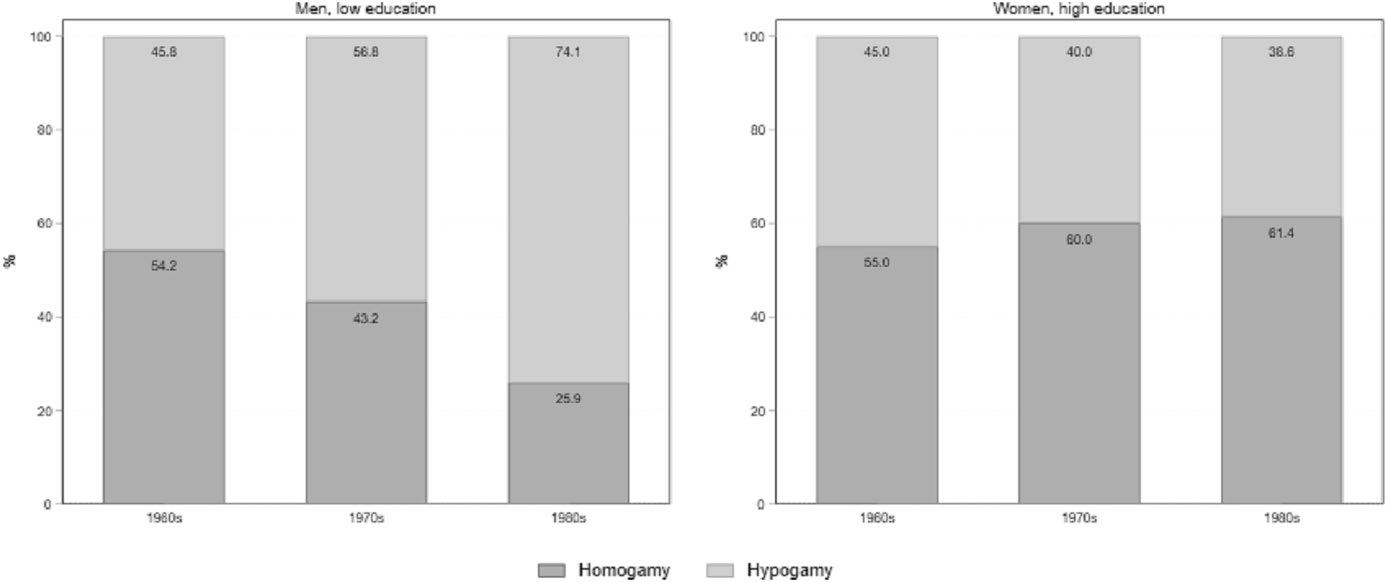
Patterns of educational assortative mating at age 34 by birth cohorts, low educated men and high educated women

Figure 4 reports patterns of educational assortative mating for the same educational groups. For low-educated men, hypogamy has become increasingly prevalent across cohorts, outnumbering homogamy starting from the generation born in the 1970s. Thus, for those men who can establish a partnership, finding an equally educated partner is becoming increasingly difficult among younger cohorts, leading them to opt for a more educated partner, and later. The pattern is not the same for highly educated women. Despite an increase in hypogamy compared to older cohorts, tertiary educated women in Germany are still more likely to establish a homogamous union rather than an hypogamous one. Thus, hypogamy seems to be stratified among groups, with less-educated men being more exposed to hypogamy than highly educated women.

### The Role of the Mating Market

After this first description, we come to the main interest of the paper, that is the role of changing mating market conditions in terms of the «new mating squeeze» due to the increasing supply of tertiary educated women for the dynamics of union formation and educational pairing across cohorts and over the life course.

**Fig. 5:**
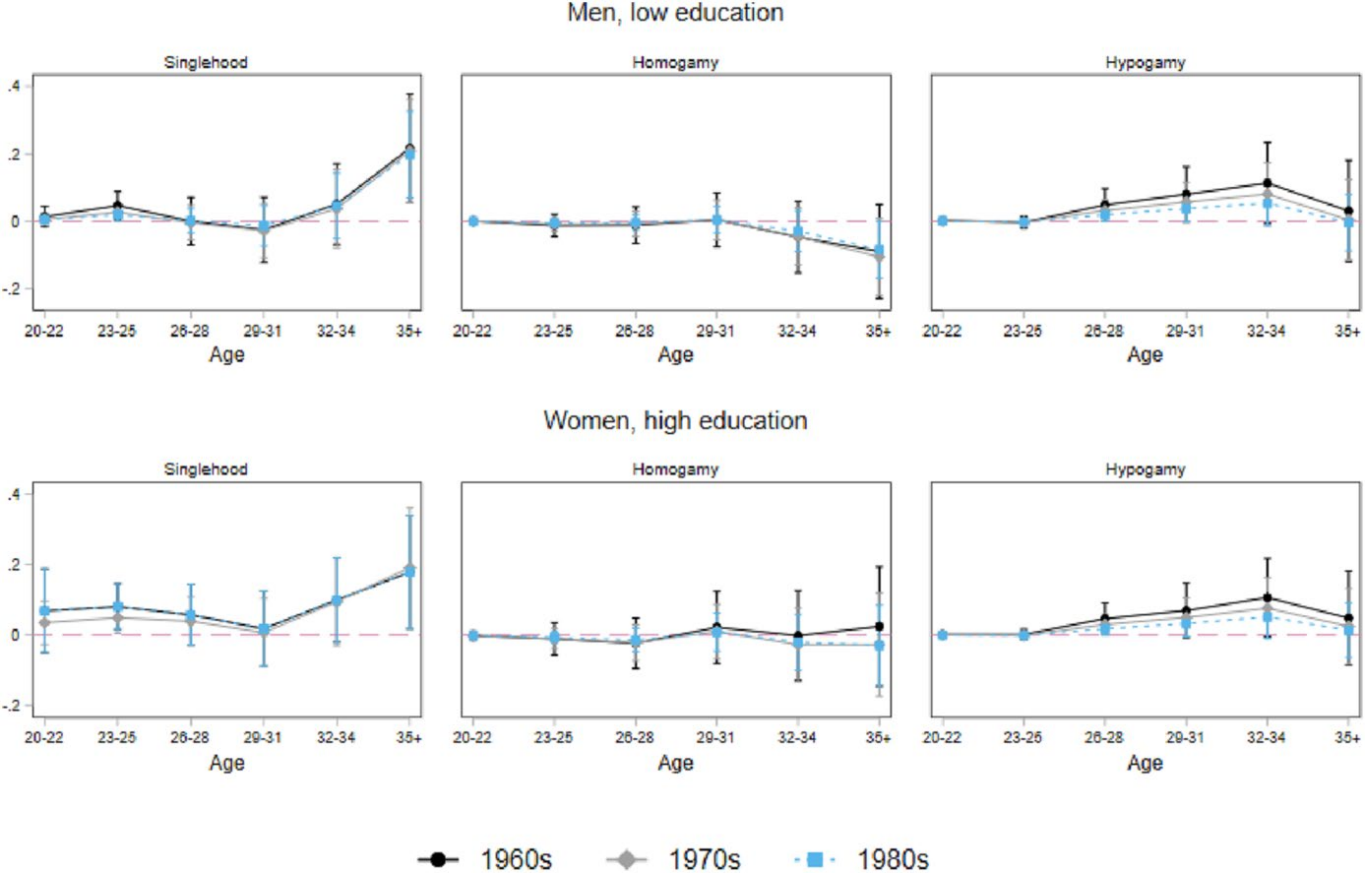
AME of the mating squeeze over the life course, low educated men and high educated women by birth cohorts

Figures 5 presents average marginal effects of the mating market squeeze on partnership formation and educational assortative mating during the partner search process for low-educated men (upper panel) and highly educated women (bottom panel) by birth cohorts. Reported results are based on multinomial discrete-time event history models, treating the outcomes jointly and allowing the mating squeeze effects to vary with age. The following states are presented: singlehood, homogamy, hypogamy (for both groups hypergamy is, by definition, not possible). The mating squeeze measure is the one from Eq. (1).

In line with the expectations, the analysis confirms the consequences of an increasing squeeze in the mating market on mating outcomes, which vary considerably over the life course. In the early stages of the partner search process—in the early twenties—the association between the conditions of the mating market and mating behaviors is negligible, meaning that when individuals are still relatively young, their mating patterns are independent of the structural constraints.

However, as people grow older, the picture changes. The turning point is around the age of thirty. From this point onwards, we find that the positive association of the mating market squeeze with hypogamy and singlehood becomes significant. In detail, for those who are still single after 34 years of age, mating market conditions have a strong impact on the likelihood of remaining single. This effect is more pronounced for highly educated women than for low-educated men. Moreover, hypogamy is positively associated with the partner market already in the mid-twenties; from age 26 onwards, partner market conditions are positively associated with the likelihood of establishing an hypogamous union both for men and women. Whereas the timing of the effect of partner market conditions is similar for both groups, low-educated men show a slightly stronger association between an increase in the advantage of women in higher education and their chances of hypogamy than highly educated women. Finally, we find that the probability of finding an equally educated partner decreases as the share of women among the highly educated increases, especially after turning 30. However, the effect of the mating squeeze is not significant.

Interestingly, these results are robust for the inclusion of regional and cohort fixed effects, as reported in Fig. 5, although effects are, as to be expected, stronger without their inclusion (see Fig. 7 in “Appendix B” for a version without cohort effects). This is important as it rules out the possibility that the findings are due to unobserved heterogeneities between regions or cultural change over cohorts. Notably, the structural constraints affect union formation almost identically for all cohorts, which gives some indirect support to the idea that changing preferences are of little relevance here. We also tested our measure of the mating market with mobile age barriers (“Appendix C”) as described above, but no relevant differences emerge. This result supports the expectations about the relation between age preferences and mating; expanding the range of potential partners could be a way to overcome structural constraints.

These results are in line with what was suggested by search theory (Oppenheimer, 1988). When the time spent in the partner market increases and the normative age for partnership formation is approaching, the costs of unfavorable mating market conditions do play a role both to the likelihood of singlehood, which means keeping on with the search, and to that of hypogamy, which reflects the choice of setting for a less conventional educational matching. An additional aspect to consider in the interpretation of our results is the changing composition of those still searching for a partner, which likely becomes increasingly negatively selected on some (unobserved) traits as people age.

**Fig. 6:**
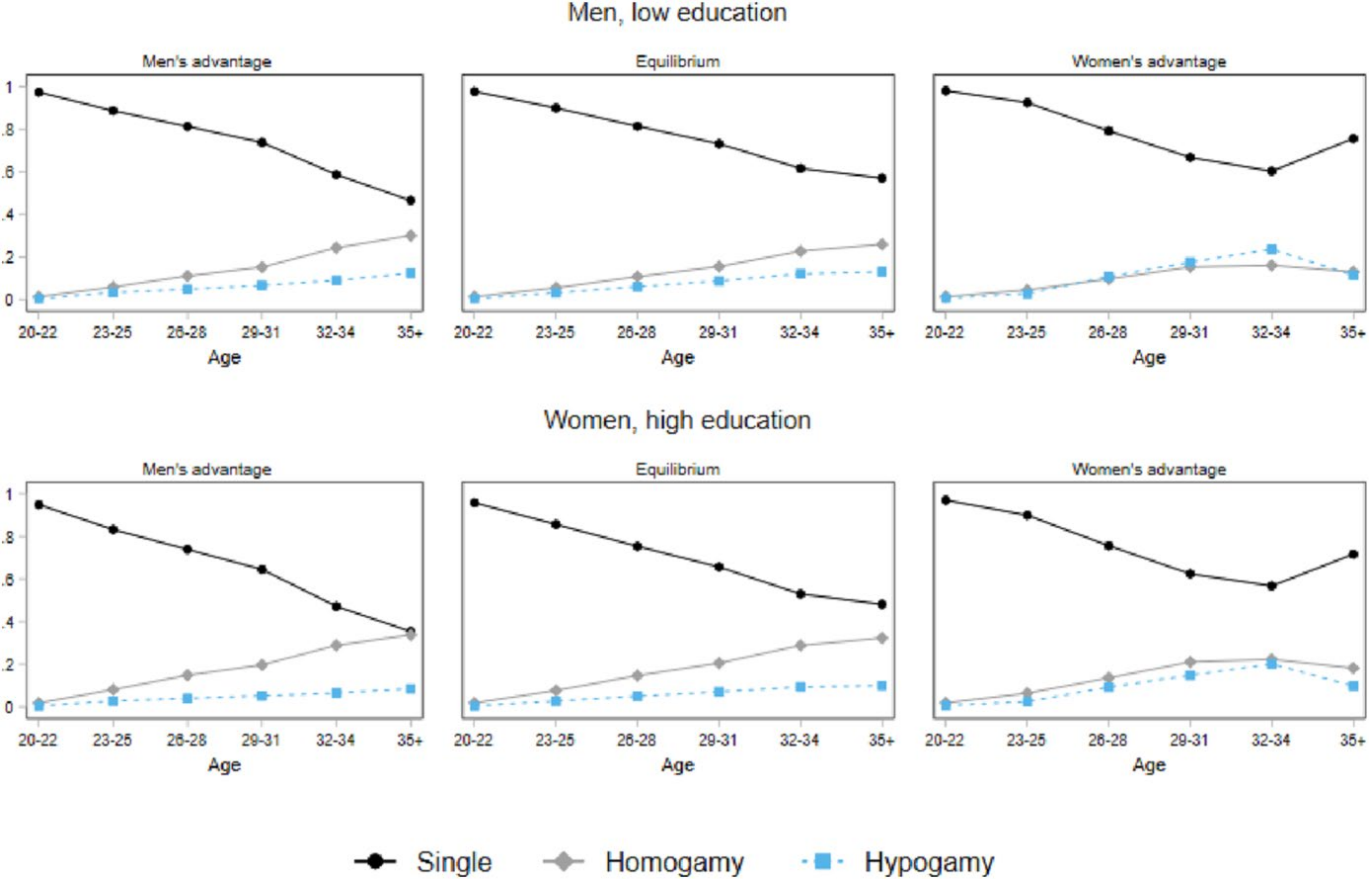
Predicted probabilities of educational assortative mating outcomes by different partner market values. Low educated men and high educated women (1971–1980)

**Fig. 7:**
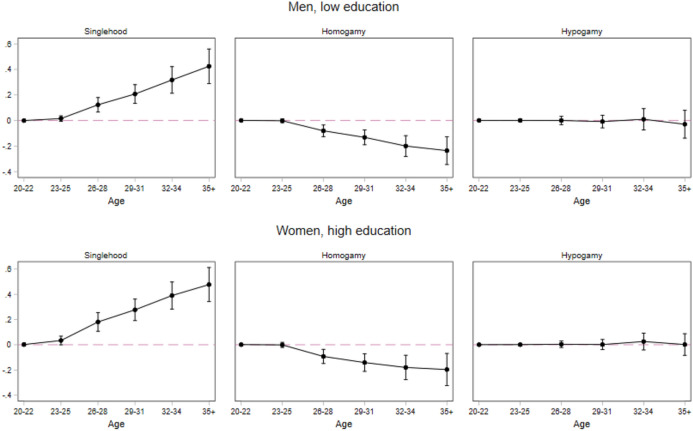
AME of the mating squeeze over the life course, low educated men and high educated women, no cohort fixed effect

To make the implications of different mating market situations for mating patterns clearer and to show different dependencies between low-educated men and highly educated women, Fig. 6 reports predicted probabilities of singlehood, homogamy, and hypogamy across ages according to different levels of the mating squeeze for the 1970s cohort. The left upper panel displays a situation of men’s advantage in higher education, with a ratio smaller than one. The right upper panel reports predicted probabilities with an equilibrium in higher education (ratio equal to one), while the last panel refers to a situation in which women have an advantage over men in higher education (ratio greater than one).

**Fig. 9:**
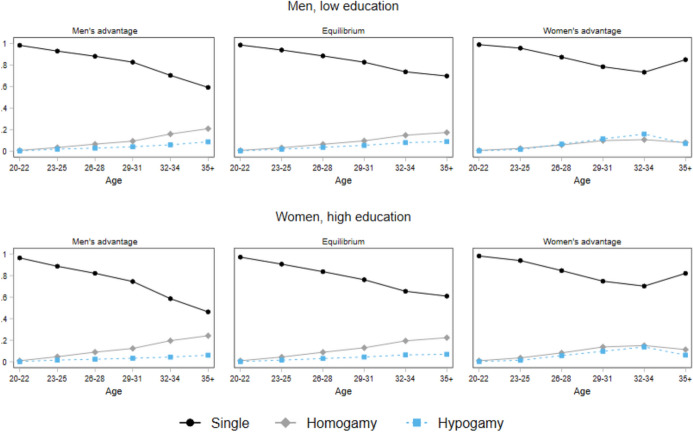
Predicted probabilities of educational assortative mating outcomes by different partner market values. Low educated men and high educated women (1981–1990)

These graphs underline the inherently dynamic nature of union formation. Further, we see how for both low-educated men and highly educated women singlehood and hypogamy become more common as the share of women with higher education increases (from the upper left panel to the right). At the same time, the likelihood of finding an equally educated partner decreases, at all ages. Moreover, for low-educated men, once women outnumber men in higher education hypogamy become more common than homogamy after the age of 30, which again proves to be a turning point. In contrast to previous findings (De Hauw et al., 2017), for highly educated women the declining probability to partner, an equally educated man with the advancement of the reversed gender gap is not completely compensated by a rise of hypogamy. Though hypogamy increases, singlehood remains the most likely outcome. Thus, highly educated women in Germany are more prone to remain single than to accept a partnership where the man has the lower education also when they get older. For the oldest cohort (born in the 1960s), the scenario is rather similar, but with singlehood being much less common. On the other hand, singlehood is more common for the youngest cohort (born after 1981, see Fig. 9, “Appendix B”).

## Discussion and Conclusion

In the twentieth century, changes in women’s roles came with a major impact on societies, including family formation. One of the most visible changes concerned the increasing involvement of women in education, to the extent that among young cohorts women today outnumber men among the tertiary educated (Buchman et al., 2008; De Hauw et al., 2014; Van Bavel, 2012; Van Bavel et al., 2018). This has, among other things, modified the structure of the partner market, generating the rise of a new education-specific mating squeeze characterized by an oversupply of highly educated women. We contribute novel findings on the relationship between changes in the mating market and partner choice and extend the literature by focusing on the timing of educational assortative mating and its relationship with structural constraints.

We observed the process of couple formation longitudinally in Germany focusing on individuals born between 1960 and 1990 for the years 1985–2018 and measured the changing partner market constraints over time at the regional level. Because an increase of women in higher education is especially detrimental for the partner market situation of highly educated women and low-educated men, who should face a shortage of equally educated potential mates, we focused on these two groups. We test the extent to which their sorting and educational pairing outcomes are associated with a partner market squeeze, and at which point of the search process this relationship becomes relevant. Following the search theory framework (Lewis & Oppenheimer, 2000; Oppenheimer, 1988), we hypothesize that partner market conditions should be particularly relevant for assortative mating not in younger ages, when individuals can prolong their partner search at affordable costs, but when they have spent years in the partner market and get closer to the normative ages of finding a partner and build a family (Wrosch & Heckhausen, 1999).

Notwithstanding some of the relevant processes occurred relatively late in Germany compared to other European countries, and the mating squeeze is not particularly pronounced yet, our descriptive evidence corroborated previous studies about changes in partnership formation (De Hauw et al., 2017; Esteve et al., 2016) and adds major details about the dynamics over the life course: younger cohorts remain single longer and hypogamous couples are becoming more common. This is true especially for low-educated men, who show a stronger pattern of disadvantage in selection into union. This first result is in stark contrast to the attention that previous literature had paid mostly to women and stresses the need to focus also on men.

We tested different measures of the mating squeeze. The relevant element appears to be the share of highly educated women over men, while the definition of the age borders and its update with age seems to be of little relevance, which confirms a mere structural interpretation. Most interestingly, the main result of our study shows how the (increase of the) local education-specific mating squeeze influences union formation, its timing, and educational sorting. As expected, the dynamics of this process change with age. Whereas in younger ages shifts in the ratio between women and men in higher education are not relevant, we find a positive association with singlehood and hypogamy as individuals grow older, especially after turning 30. This pattern is found both for low-educated men and highly educated women, but among low-educated men the likelihood to establish an hypogamous union is higher at all ages. Overall, men show a slightly larger dependency on mating market constraints in couple formation regarding their chance to establish an hypogamous union. On the other hand, structural constraints at older ages for highly educated women have a stronger association with singlehood rather than hypogamy.

Our results are partly in contrast to previous studies based on national, cross-sectional data. De Hauw et al. (2017) report a much stronger mating market squeeze effect for tertiary educated women’s chances of hypogamy than for men. These differences are likely due to the fact that we concentrate on the effects of regional changes rather than on cross-country variations in a set of European countries and follow individual-level dynamics. Our results are robust to the inclusion of regional fixed effects, placing them empirically on a more solid base.

This paper is not free from limitations and points toward further questions we cannot address here. First, Germany represents a peculiarity in the European context in the advancement of women in higher education, and it could partially help to explain our findings. Indeed, women in our sample do not suffer from a strong imbalance in their partner market, but low educated men see their chances to find an equally educated partner getting narrower. Moreover, Germany is known to be a country characterized by rather traditional gender norms that might play a role in shaping men’s educational pairings. But still in this rather unconventional setting we find interesting results. Obviously, results might change, and the effect of the partner market constraints should be reasonably stronger in other contexts where the imbalance between men and women in higher education is more pronounced. Finally, we concentrated on structural factors, leaving aside cultural aspects, which might drive women toward less educated partners earlier in their partner search. More research in this direction is needed.

In fact, we do not pretend to (and actually do not) explain changes in the couple formation process exclusively by structural changes. Many other factors might come into play (Lesthaege, 2020), and the educational composition of the mating market is only one of them. As previously argued, the mechanisms by which mating market conditions have an influence are of interest, especially after the age of 30. Following the literature, we argue that individuals adapt their preferences or requirements over time, but we deduce this from observed mating patterns. Measuring this process would be interesting, and research on the tradeoff between age, preferences, and structural conditions is needed.

## Figures and Tables

**Fig. 8:**
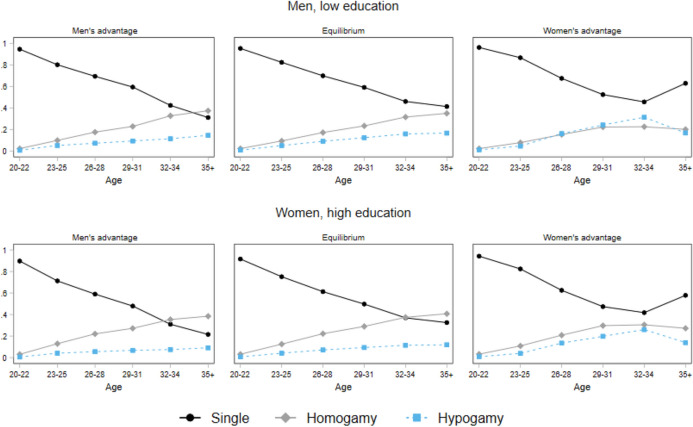
Predicted probabilities of educational assortative mating outcomes by different partner market values. Low educated men and high educated women (1961–1970)

**Fig. 10:**
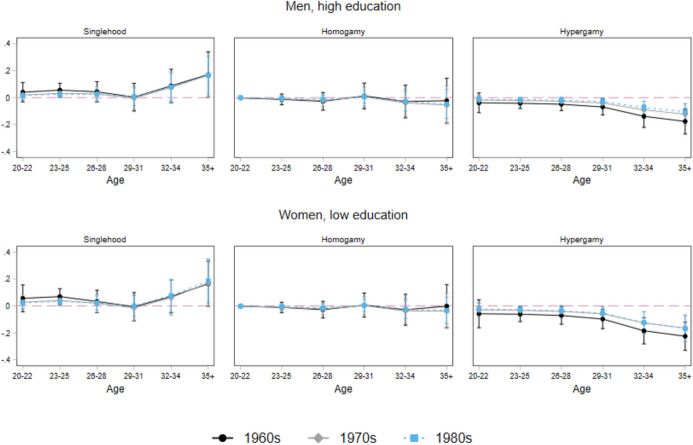
AME of the mating squeeze over the life course, high educated men and low educated women by birth cohorts

**Fig. 11:**
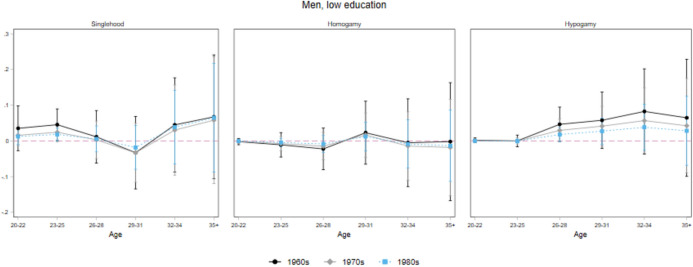
AME of the mating squeeze over the life course, low educated men (1961–1970, 1971–1980)

**Fig. 12:**
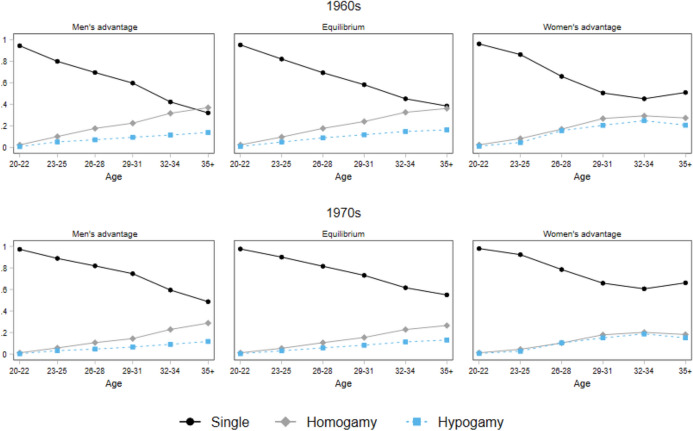
Predicted probabilities of educational assortative mating outcomes by different partner market values. Low educated men (1961–1970 and 1971–1980)

## Data Availability

The data that support the findings of this study are available from the German Institute for Economic Research, DIW Berlin for G-SOEP data and GESIS, Mannheim for Mikrozensus data, but restrictions apply to the availability of these data, which were used under license for the current study, and so are not publicly available.

## Appendix A: Sample Characteristics

See Table 1.

**Table 1:** Sample characteristics (mean and standard deviation)

	Mean (SD)
Age	30.33
	(6.79)
Birth cohort	
1960s	25.93
1970s	28.07
1980s	46.00
Sex	
Male	0.57
Female	0.43
Educational attainment	
Low	0.28
Medium	0.50
High	0.22
Born in Germany	0.74
	(0.38)
Squeeze	1.05
	(0.38)
Squeeze with mobile age barriers	1.08
	(0.37)
*N*	37,806

## Appendix B: Measures of Partner Market—Relative Advantage of Women in Higher Education

See Tables 2 and 3, and Figs. 7, 8, 9 and 10.

**Table 2:** Multinomial logistic regression coefficients (ref. single), men

	Homogamy	Hypogamy	Hypergamy
Age (ref. 20–22)			
23–25	1.567	1.552*	− 4.944***
	(0.980)	(0.810)	(1.850)
26–28	1.902**	1.784**	− 6.164***
	(0.876)	(0.795)	(1.997)
29–31	2.332***	2.460***	− 3.883**
	(0.889)	(0.888)	(1.944)
32–34	3.140***	3.035***	− 2.895
	(0.891)	(0.864)	(1.959)
35+	4.133***	4.272***	− 1.350
	(0.937)	(0.956)	(1.974)
Squeeze	− 0.253	0.142	− 5.938***
	(0.426)	(0.185)	(0.948)
Age × squeeze (ref. 20–22)			
23–25 × squeeze	− 0.297	− 0.281	4.220***
	(0.553)	(0.274)	(1.068)
26–28 × squeeze	0.116	0.373	5.937***
	(0.493)	(0.396)	(1.272)
29–31 × squeeze	0.213	0.0823	4.074***
	(0.526)	(0.580)	(1.320)
32–34 × squeeze	0.0843	0.137	3.731***
	(0.562)	(0.611)	(1.418)
35+ × squeeze	− 0.749	− 0.888	2.309
	(0.673)	(0.798)	(1.485)
Cohort (ref. 1960)			
1970	− 0.655***	− 0.543**	− 0.895**
	(0.176)	(0.271)	(0.353)
1 980	− 1.121***	− 1.179***	− 1.209***
	(0.196)	(0.304)	(0.361)
Educational attainment (ref. Low)			
Medium	0.268	− 1.030***	19.55***
	(0.184)	(0.229)	(1.783)
High	0.319	− 0.276***	0.271
	(0.198)	(0.191)	(0.219)
Region fixed effect	Yes	Yes	Yes
Constant	− 3.641***	− 4.083***	− 15.98***
	(0.920)	(1.205)	(1.858)
*N* of observations	21,441	21,441	21,441

Robust standard errors in parentheses

****p* < 0.01, ***p* < 0.05, **p* < 0.1

**Table 3:** Multinomial logistic regression coefficients (ref. single), women

	Homogamy	Hypogamy	Hypergamy
Age (ref. 20–22)			
23–25	1.573	0.667	0.648
	(1.222)	(2.512)	(2.035)
26–28	2.957**	1.305	1.822
	(1.210)	(2.495)	(2.024)
29–31	2.841**	1.123	0.996
	(1.207)	(2.489)	(2.003)
32–34	3.687***	1.709	2.450
	(1.214)	(2.498)	(2.010)
35+	3.401***	1.990	2.530
	(1.212)	(2.498)	(2.014)
Squeeze	− 0.0320	− 0.463	− 0.859
	(0.601)	(1.468)	(1.172)
Age × squeeze (ref. 20–22)			
23–25 × squeeze	0.0994	0.461	− 0.0631
	(0.643)	(1.512)	(1.251)
26–28 × squeeze	− 0.326	0.644	− 0.836
	(0.662)	(1.521)	(1.293)
29–31 × squeeze	0.0761	1.357	0.293
	(0.673)	(1.527)	(1.278)
32–34 × squeeze	− 0.369	1.187	− 0.821
	(0.699)	(1.554)	(1.316)
35+ × squeeze	0.217	1.008	− 0.702
	(0.712)	(1.572)	(1.344)
Cohort (ref. 1950)			
1970	− 0.639***	− 0.744***	− 0.640***
	(0.0782)	(0.116)	(0.128)
1980	− 1.381***	− 1.214***	− 1.072***
	− 0.639***	− 0.744***	− 0.640***
	(0.0918)	(0.133)	(0.146)
Educational attainment (ref. Low)			
Medium	0.226***	19.11	− 0.581***
	(0.0788)	(970.1)	(0.0905)
High	0.138	0.194	− 0.192
	(0.0872)	(0.970)	(0.721)
Region fixed effect	Yes	Yes	Yes
Constant	− 3.299***	− 21.51	− 0.994
	(1.182)	(970.1)	(1.954)
*N* of observations	16,365	16,365	16,365

Robust standard errors in parentheses

****p* < 0.01, ***p* < 0.05, **p* < 0.1

## Appendix C: Measures of Partner Market—Relative Advantage of Women in Higher Education with Mobile Age Barriers

See Table 4 and Fig. 11 and 12.

**Table 4:** Multinomial logistic regression coefficients (ref. single), men

	Homogamy	Hypogamy	Hypergamy
Age (ref. 20–22)			
23–25	1.818***	2.334***	0.402
	(0.445)	(0.535)	(1.014)
26–28	2.498***	2.490***	1.124
	(0.424)	(0.513)	(1.092)
29–31	2.916***	2.878***	1.237
	(0.435)	(0.548)	(1.105)
32–34	3.705***	3.576***	2.847**
	(0.452)	(0.571)	(1.122)
35+	4.355***	4.364***	3.386***
	(0.493)	(0.647)	(1.128)
Squeeze	− 0.0363	0.272**	− 0.930
	(0.147)	(0.114)	(0.635)
Age × squeeze (ref. 20–22)			
23–25 × squeeze	− 0.140	− 0.503**	0.509
	(0.235)	(0.215)	(0.649)
26–28 × squeeze	− 0.00270	0.135	0.301
	(0.250)	(0.281)	(0.776)
29–31 × squeeze	0.0747	0.196	0.671
	(0.280)	(0.352)	(0.802)
32–34 × squeeze	− 0.191	− 0.0194	− 0.509
	(0.329)	(0.430)	(0.870)
35+ × squeeze	− 0.592	− 0.552	− 0.691
	(0.402)	(0.572)	(0.877)
Cohort (ref. 1960)			
1970	− 0.212	0.295	0.132
	(0.169)	(0.262)	(0.307)
1980	− 0.779***	− 0.314	− 0.0902
	(0.178)	(0.279)	(0.312)
Educational attainment (ref. Low)			
Medium	0.129	− 0.345**	0.0418
	(0.124)	(0.162)	(0.208)
High	0.259*	− 0.578***	0.377
	(0.137)	(0.212)	(0.234)
Constant	− 4.309***	− 5.260***	− 3.898***
	(0.482)	(0.682)	(1.078)
Region fixed effect	Yes	Yes	Yes
*N* of observations	21,441	21,441	21,441

Robust standard errors in parentheses

****p* < 0.01, ***p* < 0.05, **p* < 0.1

## Footnotes

1 An additional mechanism, which we cannot test here, might be changes in the preferences of men and women, which might have shifted towards the acceptance of non-conventional couples, making these choices less costly in social terms.

2 In line with the literature (Blossfeld, 2009; Buss et al., 2001; Kalmijn, 1991; South, 1991), we implicitly assume individuals prefer partners with a similar level of educational attainment. Support for this comes from recent literature which has identified partners in hypogamous couples as negatively selected on some dimensions such as age or earnings (Chudnovskaya & Kashyap, 2020), and has shown that changes in patterns of educational assortative mating can be observed even without changes in preferences (Grow & Van Bavel, 2015).

3 MZ data are provided by GESIS for the years 1985–2015 and by European Labor Force Survey for the years 2016–2018.

4 The LFS database provides information on age by 5-years classes, so it is not possible to use exact ages. Following De Hauw et al. (2014), we estimate, for instance, the number of people between 24 and 28 taking 1/5 of individuals in the age class 20–24 and 4/5 of individuals in the age class 25–29.

5 In this work only heterosexual couples are studied.

6 For privacy reasons, MZ data are provided pooling together the smallest regions (Hamburg with Bremen and Rheinland-Pfalz with Saarland), thus making this harmonization necessary also in LFS and G-SOEP data. Given the dimensions and the geographical proximity of the regions, we do not consider it to be a big issue in our design.

## Code availability

Code for data cleaning and analysis could be provided upon request to the corresponding author.
